# *HDAC4* mutations cause diabetes and induce β‐cell FoxO1 nuclear exclusion

**DOI:** 10.1002/mgg3.602

**Published:** 2019-04-09

**Authors:** Maolian Gong, Yong Yu, Lei Liang, Dogus Vuralli, Sebastian Froehler, Peter Kuehnen, Philipp Du Bois, Jingjing Zhang, Aidi Cao, Yuantao Liu, Khalid Hussain, Jens Fielitz, Shiqi Jia, Wei Chen, Klemens Raile

**Affiliations:** ^1^ Experimental and Clinical Research Center (ECRC) a joint cooperation between the Charité Medical Faculty Max‐Delbrueck‐Center for Molecular Medicine (MDC) Berlin Germany; ^2^ Qingdao Municipal Hospital Qingdao China; ^3^ Max‐Delbrück Center for Molecular Medicine Berlin Germany; ^4^ Department of Pediatrics Anhui Provincial Children’s Hospital Hefei China; ^5^ Division of Pediatric Endocrinology, Department of Pediatrics Hacettepe University Faculty of Medicine Ankara Turkey; ^6^ Institute for Experimental Pediatric Endocrinology Berlin Germany; ^7^ Affiliated Hospital of Guangdong Medical University Zhanjiang China; ^8^ Division of Endocrinology, Department of Paediatric Medicine Sidra Medical & Research Center, OPC Doha Qatar; ^9^ German Center for Cardiovascular Research (DZHK), partner site Greifswald & Department of Internal Medicine B University Medicine Greifswald Greifswald Germany; ^10^ The First Affiliated Hospital of Jinan University Guangzhou China; ^11^ Department of Biology Southern University of Science and Technology Shenzhen China; ^12^ Department of Pediatric Endocrinology and Diabetology Charité Berlin Germany

**Keywords:** diabetes, FoxO1, *HDAC4 *mutations, pancreatic β‐cells

## Abstract

**Background:**

Studying patients with rare Mendelian diabetes has uncovered molecular mechanisms regulating β‐cell pathophysiology. Previous studies have shown that Class IIa histone deacetylases (HDAC4, 5, 7, and 9) modulate mammalian pancreatic endocrine cell function and glucose homeostasis.

**Methods:**

We performed exome sequencing in one adolescent nonautoimmune diabetic patient and detected one de novo predicted disease‐causing *HDAC4* variant (p.His227Arg). We screened our pediatric diabetes cohort with unknown etiology using Sanger sequencing. In mouse pancreatic β‐cell lines (Min6 and SJ cells), we performed insulin secretion assay and quantitative RT‐PCR to measure the β‐cell function transfected with the detected *HDAC4* variants and wild type. We carried out immunostaining and Western blot to investigate if the detected *HDAC4* variants affect the cellular translocation and acetylation status of Forkhead box protein O1 (FoxO1) in the pancreatic β‐cells.

**Results:**

We discovered three *HDAC4* mutations (p.His227Arg, p.Asp234Asn, and p.Glu374Lys) in unrelated individuals who had nonautoimmune diabetes with various degrees of β‐cell loss. In mouse pancreatic β‐cell lines, we found that these three *HDAC4* mutations decrease insulin secretion, down‐regulate β‐cell‐specific transcriptional factors, and cause nuclear exclusion of acetylated FoxO1.

**Conclusion:**

Mutations in *HDAC4 *disrupt the deacetylation of FoxO1, subsequently decrease the β‐cell function including insulin secretion, resulting in diabetes.

## 1 INTRODUCTION

About 3% of diabetic patients involve single‐gene mutations (Mendelian) that may also cause type 2 diabetes (Yang & Chan, 2016). More than twenty genes highly expressed in pancreatic β‐cells have been identified within these monogenic subtypes (Alkorta‐Aranburu et al., 2014). Recently, two national surveys revealed that most patients with monogenic diabetes are likely to be unrecognized and misdiagnosed as type 1 or type 2 diabetes (Delvecchio et al., 2017; Johansson et al., 2017). Genetic diagnosis leads to improved treatment, better prediction of disease prognosis and progression, genetic counseling, and possibly prevention. The benefit of a molecular genetic diagnosis has been shown in the patients with monogenic diabetes caused by a mutation in the *ABCC8, KCNJ11*, *HNF1A*, or *HNF4A*; all these patients were sensitive to sulfonylureas, which greatly improved glycemic control and quality of life (Hattersley & Patel, 2017). However, patients with a mutation in *GCK *typically do not require pharmacological intervention (Ajjan & Owen, 2014). Therefore, identification of novel disease‐related loci may provide further opportunities to derive new drug targets.

Histone deacetylases (HDACs) have a broad impact on the development of human disease by regulating histone modification and gene transcription (Mathias, Guise, & Cristea, 2015; Mielcarek, Zielonka, Carnemolla, Marcinkowski, & Guidez, 2015). Class II HDAC4, 5, 7, and 9 modulate endocrine cell function and glucose homeostasis in skeletal muscles, adipose tissue, and liver (Daneshpajooh et al., 2017; Lenoir et al., 2011; Mathias et al., 2015; Mihaylova et al., 2011). In addition, HDAC4 was also found to be a key regulator controlling the pancreatic β/δ lineage during embryogenesis (Lenoir et al., 2011). Makinistoglu *et al.* found that in osteoblast‐specific *Hdac4* gene‐deleted (/) mice, HDAC4 regulates not only the spatial learning and memory, male fertility, and appetite, but also glucose metabolism through its expression in osteoblasts. They observed that the insulin content and β‐cell area in the pancreas, as well as insulin levels and insulin secretion in the circulation blood, were significantly decreased in *Hdac4_osb_* / compared with the control littermates (Makinistoglu & Karsenty, 2015), indicating that HDAC4 regulates insulin content, secretion, and sensitivity through direct or indirect pathways. In humans, HDAC4 was found to be expressed in the pancreas as well, and associated with type 2 diabetes (Rani et al., 2017).

We report three pediatric hyperglycemic patients harboring individual *HDAC4* (OMIM: 605314) mutations. These *HDAC4* variants decreased the insulin secretion and down‐regulated the key β‐cells transcriptional factors of pancreatic β‐cells. Furthermore, deacetylation of FoxO1 was inhibited by these *HDAC4* mutations resulting in FoxO1 acetylation and nuclear exporting, therefore decreasing β‐cell functions.

## 2 PATIENTS AND METHODS

### 2.1 Ethical compliance

The Charité committee on human subjects’ research approved the study (EA‐No EA2/054/11) and written informed consent was obtained.

### 2.2 Patients

All individuals with hyperglycemia tested negative for β‐cell autoantibodies and developed diabetes before the age of 18 years. They were recruited from Charité and international diabetes centers. We excluded mutations in known genes causing monogenic diabetes (*GCK, HNF4A, HNF1A, HNF1B, ABCC8, KCNJ11, *and *INS*) by Sanger sequencing.

### 2.3 Variants discovery with exome sequencing and Sanger sequencing

We conducted exome sequencing in one patient and his healthy parents and analyzed the sequencing results with our reported pipeline (Kuhnen et al., 2014) for rare de novo variants (Table S1) against the available databases using the Agilent SureSelect Human All Exon Kit (Agilent SureSelect v4, 50 Mb) and next‐generation sequencing (Hiseq, Illumina, USA). We confirmed the *HDAC4* (NG_009235.1) mutation (p.H227R) by Sanger sequencing only in this patient, but not in his parents and other 200 healthy controls (Applied Biosystems, USA). In addition, through screening our diabetic cohort of 94 patients with the unknown origin for *HDAC4* mutations (Table S2), we detected two other individual heterozygous *HDAC4* mutations in two unrelated families (p.D234N & p.E374K).

### 2.4 Mutagenesis and transfection

The human Flag‐tagged *HDAC4 *cDNA (pcDNA3 N‐Flag) was kindly provided by Prof. Jens Fielitz of Charite. We sequenced the cDNA clones before the experiments, confirming the HDAC4 cDNA and the tagged Flag sequences. We performed mutagenesis with the primers (Table S2) designed for all the three mutations (p.H227R, p.D234N, p.E374K) according to the manufacturer's instruction (QuikChange II Site‐Directed Mutagenesis Kit, Agilent Technologies, USA). We extracted plasmid DNA using Qiagen kits: QIAprep® Spin Miniprep Kit (minipreps) and QIAfilterTM Plasmid Maxi Kit (Maxipreps) according to the manufacturer's instruction (QIAGEN, Germany). We transfected the HDAC4 constructs and the empty vector into the SJ pancreatic β‐cells (Jia et al., 2015) using AmaxaTM Cell Line NucleofectorTM Kit V and AmaxaTM NucleofectorTM 2b device with transfection efficiency of 80%–90% (Lonza, Switzerland, Figure S1a), and transfected the HDAC4 constructs into MIN6 cells with lipofectamine 2000 reagents with transfection efficiency of 70% or more (#11668027, Thermofisher, USA, Figure S1b). All the constructs were screened with Sanger sequencing to ensure of the only pointed mutations before further investigation.

### 2.5 Insulin secretion assay

The glucose‐sensitive mouse β‐cell line SJ β‐cells were cultured and insulin‐secretion assay was performed as reported earlier (Jia et al., 2015). In brief, insulin secreted into medium and from whole cell lysates was quantified with Mouse High Range Insulin ELISA kit, according to the manufacturer's protocol (Alpco, USA). Cells were incubated for 30 min with low (3.3 mmol/L) or high (16.7 mmol/L) glucose concentration. We normalized secreted insulin by the total cellular protein. All the values were expressed in relation to cells transfected with wild‐type human HDAC4.

### 2.6 Quantitative RT‐PCR

We checked the mRNA expression levels of key markers for pancreatic β‐cells using SYBR® Green chemistry (Applied Biosystems, USA) with the according primers (Table S3). Key factors for β‐cells function and maintenance include Pdx1, Neurod1, Foxa2, Hnf1α, Hnf1β, Hnf4α, Nkx6.1, MafA, FoxO1, Scl2a2, Abcc8, Kcnj11, Ins1, Ins2, and Gck.

### 2.7 Immunoblotting (IB), immunofluorescence (IF), and immunoprecipitation (IP)

Immunoblotting and immunofluorescence staining were carried out as previously described (Jing et al., 2011). Immunoprecipitation was performed using Dynabeads Protein A Immunoprecipitation Kit according to the manufacturer's protocol (#10006D, Thermofisher, USA). Antibodies used in this study: HDAC4 (#7628, Cell signaling, Germany), FoxO1 (#2880, Cell signaling, Germany), Pdx1 (sc‐14664, Santa Cruz, USA), acetylated lysine (#9441, Cell signaling Germany), α‐Tubulin (#T6199, Sigma, USA), and total histone H3 (#4499, Cell signaling, Germany) (Table S4).

We used GraphPad Prism 5.0 software (GraphPad Software). Differences between three or more groups were assessed by 1‐way ANOVA with Dunnett's correction. *p *< 0.05 were considered significant.

## 3 RESULTS

### 3.1 Patients with defective insulin secretion harbor *HDAC4* mutations

**Figure 1:**
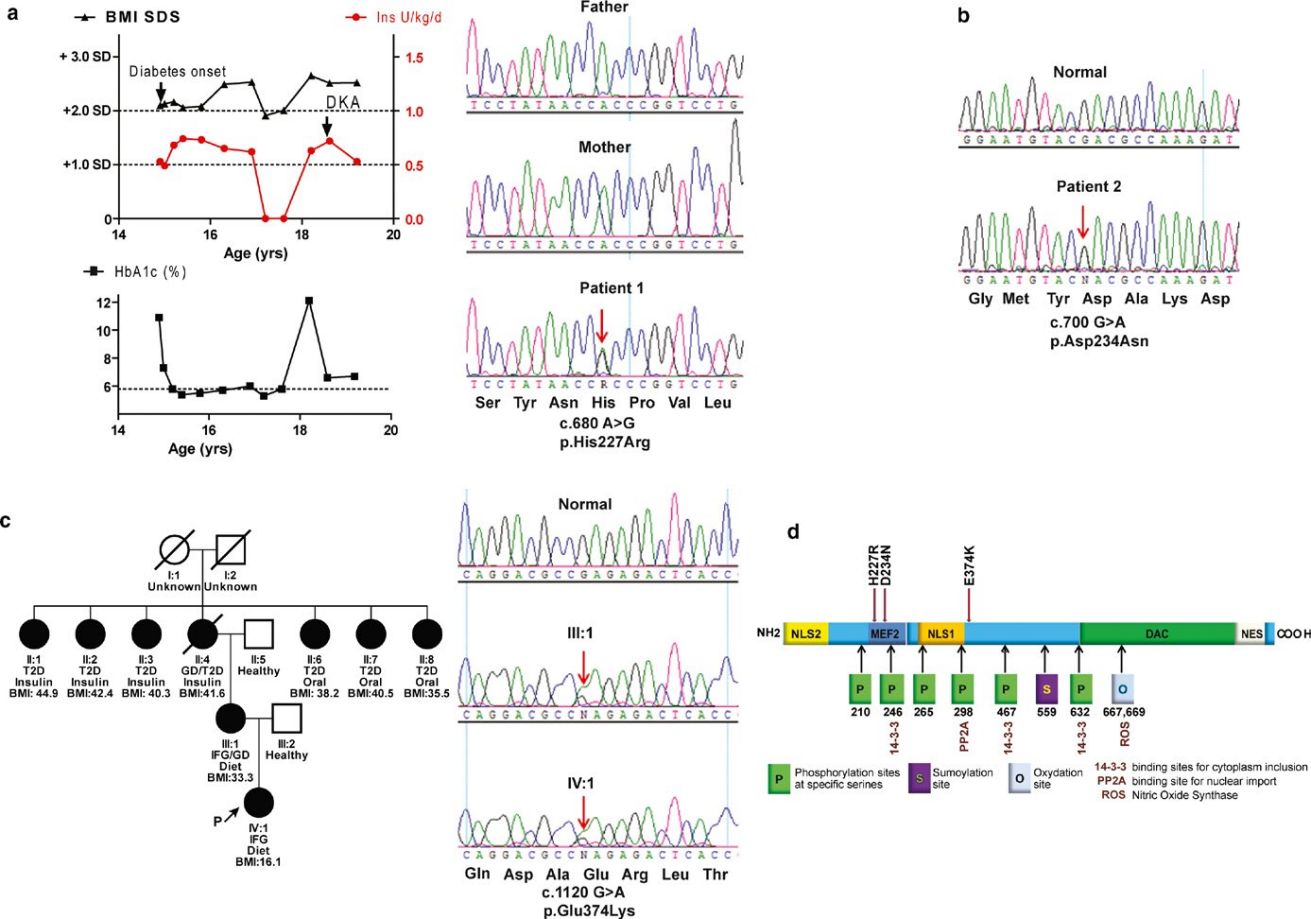
Mutations in *HDAC4 *are present in pediatric hyperglycemic patients. a. The disease progress of the first patient and the de novo *HDAC4* mutation (p.H227R) found in the affected patient. DKA: *diabetic ketoacidosis*. b. Sporadic patient 2 carried one heterozygous mutation (p.D234N) in *HDAC4. *c. The studied Turkish family presented as early onset of diabetes (age 30–40 years) and obesity carried one heterozygous mutation (p.E374K) in *HDAC4*. Solid symbols illustrate affected individuals, open symbols illustrate unaffected individuals. Squares illustrate male subjects and circles illustrate female subjects; a diagonal line through a symbol represents a deceased individual; arrow indicates the index patient. Under the symbols: diagnosis, treatment, and body mass index (BMI) of the affected individuals were shown vertically. T2D: type 2 diabetes, GD: gestational diabetes, Oral: oral drug administration, IFG: impaired fasting glucose d. Schematic representation of the HDAC4 protein domains and its established binding sites (MEF2s), nuclear localization signal (NLS1, NLS2) in the N‐terminal part; a highly conserved deacetylase domain, a nuclear export signal (NES) at the C‐terminal end, three 14‐3‐3 binding sites, and other regulatory sites (phosphorylation, Sumoylation or oxidation sites, binding sites for nuclear import, as well as nitric oxide synthase). The locations and character of the *HDAC4* mutations, modified from Mielcarek et al (Mielcarek et al., 2015)

Our first patient developed a large pubic abscess at the age of 15 years. Hyperglycemia and elevated HbA1c were noticed (Figure 1a, left and Table 1). He received multiple daily insulin injections but discontinued insulin after an intended weight loss (Figure 1a, left). Insulin was then reinstituted after an increase in HbA1C. At the age of 19 years, he skipped insulin injections and developed ketoacidosis (Figure 1a, left and Table 1).

**Table 1:** Clinical characteristics of patients with heterozygous *HDAC4* mutations

Patient ID	Patient 1	Patient 2	Patient 3
Gender	Male	Male	Female
Ethnicity	German	German	Turkish
Family history	Parents healthy	Unknown	Mother diabetes (diet), grandmother mother side diabetes (insulin)
Diabetes onset (years)	14.9	8.6	6.3 (IFG)
Initial glucose (mg/dl)	293 (random)	>400 (random)	110 (fasting)
Initial HbA1c (%)	10.4	11.0	5.9
HOMA‐IR	n.a.	n.a.	2.6 (elevated for prepubertal)
BMI (kg/m^2^)	30.1	18.2	16.1
BMI‐SDS	+2.3	+0.6	0.48
Antibodies^a^	Negative	Negative	Negative
Diabetes treatment	Initial MDI insulin, off insulin age 17.2–18.4, continued NPH insulin alone	Insulin (pump), no remission	Diet
g.Pos (Hg19)	2:240078401 T>C	2:240078381 C>T	2:240056115 C>T
c.pos^b^	c.680A>G	c. 700 G>A	c. 1120 G>A
Zygosity	Heterozygous	Heterozygous	Heterozygous
Ref.SNP (MAF)	Novel	rs549858743 (0.0002)^c^	rs370963321 (3.13e−5)
Function change	p.H227R	p.D234N	p.E374K
Prediction^d^	Disease causing	Disease causing	Disease causing

Note

AA, amino acid: MAF, minor allele frequency from ExAc database; NA, not available; IFG, impaired fasting glucose; g.Pos, genomic position; c.Pos, coding position.

a Antibodies tested: IA‐2, GAD65, IAA.

b NM_006037.3.

c It is novel in the European population.

d Classified as pathogenic in Polyphen, SIFT, Mutation Taster, GERP++, and LRT.

We performed exome sequencing in the patient and his healthy parents. From the 34 de novo heterozygous variants, eight variants were functionally predicted to be disease causing by multiple in‐silico predictive algorithms (Table S1). We focused on *HDAC4* due to its specific expression pattern and roles in pancreatic islet development (Lenoir et al., 2011), gluconeogenesis, and insulin resistance (Mathias et al., 2015; Mihaylova et al., 2011). We confirmed the novel heterozygous HDAC4 mutation solely in the patient (Hg19, chr2: 240,078,401, T > C, H227R > p.H227R, novel in the gnomAD database, Figure 1a, right, and Table 1), but not in his parents and in additional 200 healthy controls. The mutation was not observed in 138,496 alleles in the gnomAD database and is predicted to probably damage the protein structure, function, protein–protein interaction, or to interfere with the conservation with multiple lines of computational algorithms (Table 1). Therefore, this variant is likely pathogenic to the glucose metabolism according to the American College of Medical Genetics and Genomics (ACMG) guideline of the sequence variants (Richards et al., 2015). After screening our cohort of 94 patients for *HDAC4* mutations (Table S2), we identified two more *HDAC4 *mutations in two unrelated hyperglycemic individuals (Figure 1b,c**), **these two variants were not detected in the 200 healthy individuals either.

The second patient had hyperglycemia (> 400 mg/dl) and a highly elevated HbA1c level (Table 1) at the age of 8.6 years. Multiple daily insulin injections were begun. He carried an *HDAC4 *mutation (Hg19: chr2: 240078381, G>A. p.H234N, rs549858743, MAF in the European population: 0/126708 in the gnomAD database, Figure 1b), which was not found in any other patients of our cohort. Unfortunately, his parents were not available for genetic testing.

The third patient came from a large Türkish kindred and was investigated because of high diabetes prevalence in this family and her persistent fasting hyperglycemia. She exhibited slightly increased fasting glucose (110–120 mg/dl) and HbA1c levels when she was 6 years old (Table 1). Her now 33‐year‐old mother was obese (BMI: 33.3 kg/m^2^), had increased fasting glucose (110–120 mg/dl) since puberty, gestational diabetes during pregnancy, but was managed since then with diet alone (Figure 1c). The maternal grandmother had gestational diabetes that progressed to type 2 diabetes at the age of 32 years. She had required insulin and died at the age of 49 years from myocardial infarction. Overall, six sisters of the maternal grandmother were diagnosed with type 2 diabetes at ages between 30 and 40 years, of whom three had been treated with insulin (for 3–10 years) and three were treated with oral antihyperglycemic drugs. All were obese (BMI: 35–45 kg/m^2^). We found one heterozygous *HDAC4* mutation in this family (Figure 1c and Table 1), which is relatively rare in the European population (Hg 19: chr2: 240056115 G>A, E374K>p.E374K, rs370963321, MAF in the European population: 1/27,424 in the gnomAD database).

All three detected variants localize at the N‐terminal region of the HDAC4 protein, which harbors cleavage and phosphorylation sites (Mielcarek et al., 2015), suggesting this region as an important regulatory HDAC4 domain (Figure 1d).

### 3.2 HDAC4 mutations disrupt insulin secretion and down‐regulate transcription factors

**Figure 2:**
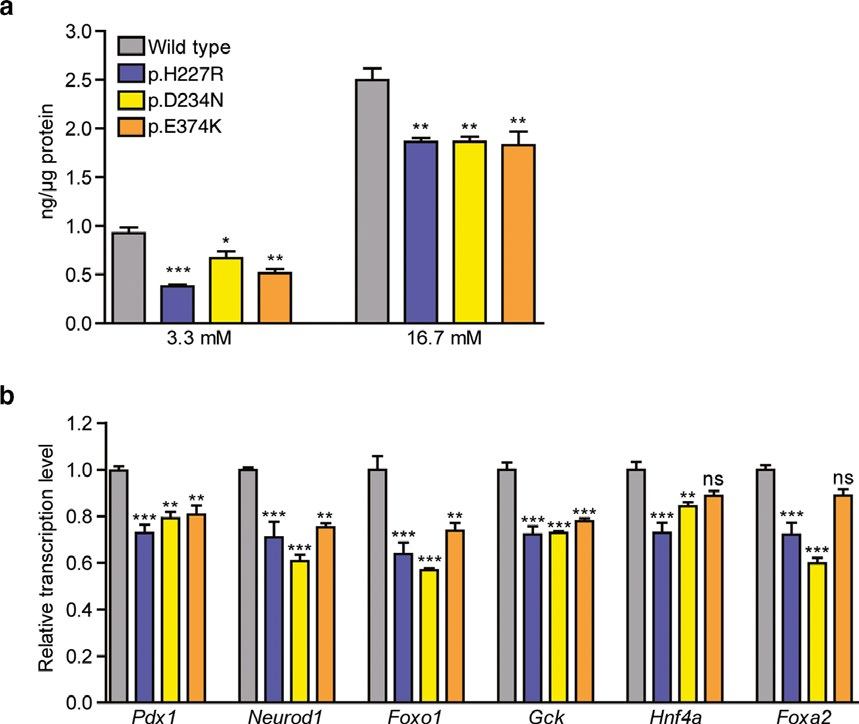
*HDAC4* mutations impair β‐cell function. a. Insulin secretion in mouse SJ β‐cells was suppressed by transfection with three mutated *HDAC4* at both low (3.3 mmol/L) and high glucose levels (16.7 mmol/L) compared to that in the cells transfected with wild‐type *HDAC4*. Technical replications =4, biological replications =4. b. Pancreatic β‐cell‐specific transcriptional and regulatory factors were down‐regulated by the mutated *HDAC4*. Statistical analyses were performed using GraphPad Prism 5.0 software. *: *p* < 0.05, **:*p* < 0.01, ***: *p* < 0.001, ns: not significant

We transfected wild‐type and the three mutated *HDAC4 *constructs into glucose‐sensitive mouse SJ β‐cells (Jia et al., 2015). Insulin secretion was significantly decreased by the three *HDAC4* mutations compared to wild‐type *HDAC4* in both low (3.3 mmol/L) and high glucose (16.7 mmol/L) levels (Figure 2a). To examine the effects of each of the *HDAC4 *mutations on the expression of key factors in β‐cells (Table S3), we performed quantitative RT‐PCR and found that expression of *FoxO1, Pdx1, Neurod1*, and *Gck *was significantly down‐regulated by each of the three *HDAC4* mutations, compared to wild‐type *HDAC4 *(Figure 2b). For *Hnf4a* and *Foxa2,* down‐regulation could only be achieved by the two *HDAC4 *p.H227R and p.D234N mutations, but not by the p.E374K mutation, corresponding to the milder phenotypes in the third patient’s family. The regulation of the transcription factors of *FoxO1, Pdx1, Neurod1*, and glucokinase, *Gck, *by the *HDAC4* mutations were repeatedly detected irrespective of transfection times (48 and 72 hr) or cell batches.

### 3.3 *HDAC4* mutations inhibit nuclear FoxO1 translocation

**Figure 3:**
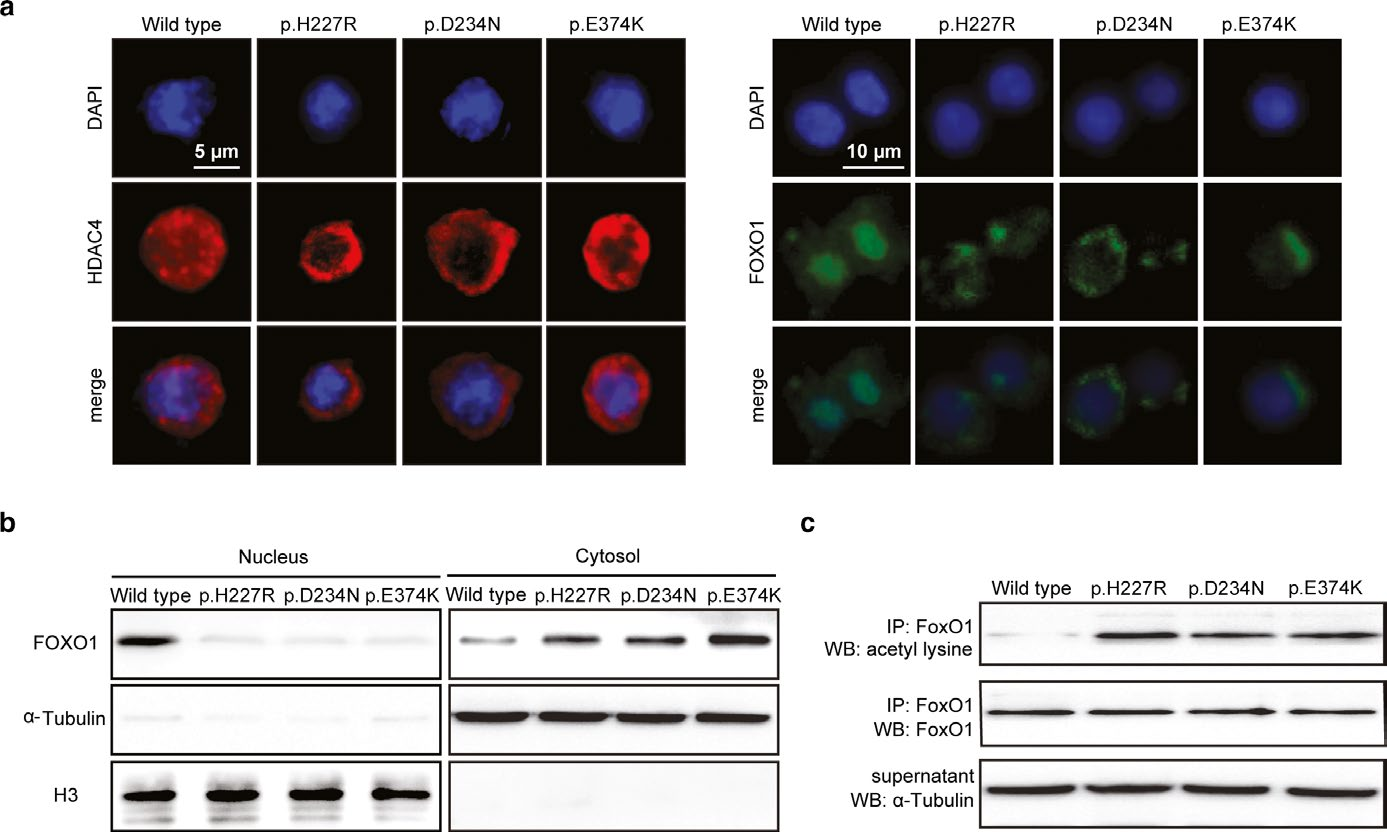
*HDAC4* mutations export FoxO1 from nuclear to cytosol through increasing levels of acetylation in Min6 cells. a. Immunostaining of DAPI (blue), HDAC4(red), and FoxO1 (green) in MIN6 cells that were transfected with wild‐type (WT) or three mutated *HDAC4* (p.H227R, p.D234N, and p.E374K). Both HDAC4 (left) and FoxO1 (right) were translocated into the cell cytosol in the mutated *HDAC4 *transfected cells compared to the wild type of *HDAC4 *transfected cells. b. Immunoblotting with antibodies against FoxO1, α‐Tubulin, and Histone 3 (H3) of lysates separately extracted from nuclear (left) and cytosol (right) of MIN6 cells that were transfected with wild‐type (WT) or three mutated *HDAC4* (p.H227R, p.D234N and p.E374K). c. Immunoblotting with antibodies against acetylated‐lysine FoxO1, and α‐Tubulin using anti‐FoxO1 immunoprecipitation lysates of MIN6 cells that are transfected with wild‐type (WT) or three mutated *HDAC4* (p.H227R, p.D234N, and p.E374K)

FoxO1 is translocated into the nuclear when deacetylated by SIRT1, induces the *NeuroD1* and *MafA* expression and thus protects against pancreatic β failure (Kitamura et al., 2005). To investigate whether or not cellular translocation of FoxO1 is critically influenced by our diabetes‐associated *HDAC4* mutations, we transfected wild‐type and three mutated *HDAC4s* (p.H227R, p.D234N, p.E374K) into mouse MIN6 β‐cells and examined FoxO1 with immunostaining. We found that the three *HDAC4* mutations promoted both HDAC4 and FoxO1 nuclear exclusion, compared to wild‐type *HDAC4* (Figure 3a). To further validate the FoxO1 cellular translocation, we separated cytosol proteins from the nuclear in MIN6 cells. Subsequently, immunoblotting specifically demonstrated that all three *HDAC4* mutations prevented translocation of FoxO1 from a cytoplasmic into a nuclear fraction in the mouse β‐cells (Figure 3b). The experiments were replicated with different transfection time as well as with different batches of MIN6 and SJ β‐cell lines (Figure S2a,b), respectively. However, the PDX1 cellular location was not affected by the *HDAC4* mutations in pancreatic β‐cells (Figure S2c).

### 3.4 *HDAC4* mutations specifically fail to deacetylate FoxO1 in pancreatic β‐cells

Acetylated FoxO1 is primarily cytoplasmic, when fully deacetylated, it lingers in the nuclear and protects β‐cell function (Kim‐Muller et al., 2016). To investigate whether or not the *HDAC4* mutations fail to deacetylate FoxO1, we performed immunoprecipitation with specific antibodies against FoxO1 and measured acetylated‐lysine level by western blot analysis. Interestingly, we observed that acetylated FoxO1 dramatically increased if cells were exposed to diabetes‐associated *HDAC4 *mutations, compared to wild‐type *HDAC4*, while there was no much change in total FoxO1 protein level (Figure 3c). These results indicate that FoxO1 is a deacetylation target of HDAC4 and diabetes‐associated *HDAC4* mutations show a selective impaired ability to deacetylate FoxO1 in pancreatic β‐cells.

## 4 DISCUSSION

We detected a de novo *HDAC4 *p.H227R variant by exome sequencing in a diabetic child and afterward detected two young hyperglycemic individuals harboring independent *HDAC4 *variants (p.D234N, p.E374K). Though the latter two variants have been shown in the population of Asian in both gnomAD and ExAC databases (p.D234N: 13/18870 in East Asian, 17/277220 in all populations; p.E374K: 139/30660 in South Asian; 149/242078 in all populations), the frequencies of which are quite rare in the European population (In non‐Finnish European D234N: 0/126708; p.E374K: 4/109696), which are very likely due to population heterogeneity or the possibility of incomplete penetrance. The incomplete penetrance for severe Mendelian childhood disorders is likely more common than previously believed, even with homozygous variants in the candidate genes with strong confidence for the relevant disorders (Chen et al., 2016). This state‐of‐affair could be due to the combination of factors, such as expression patterns of normal alleles, epigenetic modification, genetic drift, and specific population genetic variant modifiers, or environmental modifiers (Cooper, Krawczak, Polychronakos, Tyler‐Smith, & Kehrer‐Sawatzki, 2013).

The utility of publicly available databases such as gnomAD or ExAC as controls has limitations since both these databases contain many type 2 diabetes cohorts (http://gnomad.broadinstitute.org/about). Most cases of monogenic diabetes were often misdiagnosed as having type 2 or type 1 diabetes (Delvecchio et al., 2017; Johansson et al., 2017). Furthermore, Mendelian diabetes mutations were previously thought to be highly penetrant, but were found to have reduced or incomplete penetrance, and therefore can be identified more frequently in age‐matched normoglycemic individuals compared with other highly penetrant mutations (Patel et al., 2017). The reduced penetrance of the mutation is important to genetic counseling of at‐risk relatives undergoing predictive tests because the chance of presenting with diabetes is no longer based simply on the odds of inheriting the mutation. Not all heterozygous persons will develop diabetes, and those that do are likely to have a later onset. Therefore, individuals harboring the specific mutation could be encouraged to perform preventive strategies such as healthier lifestyle. We have a similar scenario of our third hyperglycemia family. The index child showed slight hyperglycemia; her mother and mother‐side grandmothers all showed either gestational diabetes and/or early‐onset type 2 diabetes. Even though the *HDAC4 *p.E374K variant in this family has a relatively high allele frequency of the South Asian, the allele frequency in European is quite rare, indicating both population heterogeneity and the incomplete penetrance of this variant to the hyperglycemia.

Our functional analysis revealed that the three detected *HDAC4 *variants could disrupt the mature β‐cells to secrete insulin in both statuses of basic and high glucose concentration. The decreased β‐cell function was due to the down‐regulation of the vital transcriptional or glucose transport factors by the *HDAC4 *variants. So far, there are only a few studies focusing on the association for HDAC4 and the function of the pancreas and/or islets (Lenoir et al., 2011; Makinistoglu & Karsenty, 2015). During embryogenesis, overexpression of HDAC4 and HDAC5 showed a decreased pool of insulin‐producing β‐cells and somatostatin‐producing δ‐cells (Lenoir et al., 2011). In contrast, the osteoblast‐specific Hdac4 knock‐out mice showed decreased β‐cell area as well as insulin contents in the pancreas, and decreased insulin secretion in the blood (Makinistoglu & Karsenty, 2015). Recently, *HDAC4* was found to be one of the protective genes for type 2 diabetes in human (Rani et al., 2017).

How HDAC4 operates to influence insulin secretion in the pancreas is a question that requires further detail investigation. From this report, we showed that the post‐transcriptional regulation (deacetylation) of FoxO1 was affected by the variants of *HDAC4* in patients, the endogenous HDAC4 expression and its enzyme activity remained unchanged (data not shown). The HDAC4 variants, therefore, very likely cause the FoxO1 deacetylation failure, results in FoxO1 acetylation and nuclear exclusion, therefore, disrupts β‐cell function and maintenance (Figure 3). The diverse severity of clinical features; from severe hyperglycemia (p.H227R & p.D234N) to the modest IFG (p.E374K) matches their responding functional abnormality from different variants (Figure 2ab). All the above‐mentioned evidence supports that these pathogenic variants cause hyperglycemia according to the guideline of The American College of Medical Genetics and Genomics (ACMG) (Richards et al., 2015).

*HDAC4* haploinsufficiency was reported to result in a severe brachydactyly mental retardation syndrome. The syndrome includes a variety of clinical features, such as brachydactyly E, developmental delays, behavioral problems, and obesity in 80% of the patients. However, the glucose levels were not reported (Williams et al., 2010). Mihaylova *et al.* found that HDAC4/5 recruit HDAC3 resulting in promoter induction of gluconeogenesis enzymes such as G6Pase, controls the FOXO acetylation in hepatocytes and liver; contribute to the hyperglycemic phenotype of type 2 diabetic rodent models in their insulin‐resistant states (Mihaylova et al., 2011). Similarly, in skeletal muscle cells, HDAC4 down‐regulates genes involved in energy expenditure, results in insulin resistance and type 2 diabetes (Fang et al., 2016). Moreover, Hdac4 suppressed transcription of Glut4, a key protein for glucose uptake in adipocytes (Henriksson et al., 2015), and disruption of *Hdac4 *in macrophages is sufficient to promote insulin resistance and obesity (Luan et al., 2014). From this report, obesity is present in two of the three hyperglycemic families, and the detected *HDAC4* variants are missense variants instead of HDAC4 haploinsufficiency. Our first patient was severely obese and his glucose levels were improved with the loss of weight. For the third family, the mother and seven mother‐side grandmothers of the index patient are present as severe obesity and type 2 diabetes, even though the index patient is currently slim very likely due to the young age, she showed slightly insulin resistance (Table 1). The pathogenic roles of HDAC4 in hyperglycemia, and/or, obesity should be thus systematically investigated in the near future.

Except for its critical regulatory role of insulin signaling in liver (Langlet et al., 2017), FoxO1 maintains β‐cell mass (Kitamura et al., 2002; Okamoto et al., 2006), β‐cell identity, and proper β‐cell function (Kawamori et al., 2006; Talchai, Xuan, Kitamura, DePinho, & Accili, 2012). Impaired FoxO activity causes MODY‐like diabetes through the reduced metabolic flexibility of β‐cells (Kim‐Muller et al., 2014). FoxO1 transcriptional activity is regulated by a complex array of post‐translational modifications, one of the key events in regulating FoxO1 activity in β‐cells is the acetylation of specific lysine residues. When fully acetylated, FoxO1 exports from nuclear to the cytoplasm, whereas fully deacetylated, it imports into the nuclear and increases its transcriptional activity. In addition, the acetylation of FoxO1 increases the levels of its phosphorylation, resulting in its cytoplasm translocation and down‐regulation of the expression of the target genes (Matsuzaki et al., 2005).

Deacetylated FoxO1 protects β‐cell function by limiting mitochondrial lipid utilization through decreasing fatty acid oxidation and enhancing insulin secretion in the context of diabetes (Kim‐Muller et al., 2016); which is in consist in the findings that the levels of acetylated FoxO1 are markedly increased in pancreatic tissues of diabetic, compared to healthy rats (Ding et al., 2014). We found elevated levels of acetylated FoxO1 in the pancreatic β‐cells exposed to the *HDAC4* mutations compared to the wild type, indicating the failure of FoxO1 deacetylation in the diabetic patients. (Zhang et al., 2016) revealed that FoxO1 plays an important role in regulating β‐cell compensation for insulin resistance to maintain euglycemia in obesity; FoxO1 acts in parallel with Pdx1 in nuclear of β‐cells and colocalize in human fetal islets, in contrast to the assertion that FoxO1 counteracts Pdx1 by promoting Pdx1 nuclear exclusion in β‐cells (Kawamori et al., 2006). In this report, we found dramatically increasing levels of acetylation FoxO1 and obvious nuclear export of both HDAC4 and FoxO1, but the location of Pdx1 remains unchanging, indicating the *HDAC4* mutations disrupt the deacetylation of FoxO1, but not the pdx1. We therefore hypothesize FoxO1 is deacetylated not only by Sirt1, Sirt2, and Sirt6, but also by HDAC4 in the pancreas (Daitoku et al., 2004; Kim‐Muller et al., 2016; Kitamura et al., 2005; Song, Wang, Ka, Bae, & Park, 2016). The regulation of FoxO1 to β‐cell compensation to overnutrition and obesity (Zhang et al., 2016) was thereafter disrupted by the FoxO1 deacetylation failure in the diabetic patients harboring *HDAC4* mutations. How these mutations cause deacetylation failure of the FoxO1 and further affects the β‐cell function changing should be investigated in the subsequent studies.

In summary, we detected three *HDAC4 *variants (de novo*,* and with incomplete penetrance) in three separate hyperglycemic families with/without obesity. In pancreatic β‐cells, these variants were found to decrease the insulin secretion and down‐regulate the pancreatic β‐cells key factors. In the mechanism, the β‐cells dysfunction might be caused by the FoxO1 deacetylation failure due to the *HDAC4* mutations. The functional evidence in combination with what was reported in previous animal model studies: the FoxO1 deacetylation affects the function of β‐cells, clearly shows that the *HDAC4* mutations are likely pathogenic of childhood hyperglycemia, and the *HDAC4 *variants cause childhood hyperglycemia with at least limited or even moderate evidence (Strande et al., 2017). Our study also suggests that HDAC4‐mediated FoxO1 deacetylation could represent a novel target for pharmacotherapy, not only of decreasing glucose‐sensitive insulin secretion but also for insulin resistance. However, the complex interplay of histone modifications and hyperglycemia is required for further comprehensive investigation.

## CONFLICT OF INTEREST

The authors have no interest conflicts.
