# Supplementary material for: HDAC4 mutations cause diabetes and induce β‐cell FoxO1 nuclear exclusion
Source: Mol Genet Genomic Med. 2019 Apr 9;7(5):e602. doi: 10.1002/mgg3.602 (PMC6503015; doi:10.1002/mgg3.602)
Supplement: Supplementary file 1 [file MGG3-7-e602-s001.docx]

**Online Supplementary Materials**

***HDAC4* mutations cause diabetes and induce β**-**cell FoxO1 nuclear exclusion**

Maolian Gong, Yong Yu, Lei Liang, Dogus Vuralli, Sebastian Fröhler, Peter Kühnen, Philipp Du Bois, Jingjing Zhang, Aidi Cao, Yuantao Liu, Khalid Hussain, Jens Fielitz. Shiqi Jia, Wei Chen, Klemens Raile

**
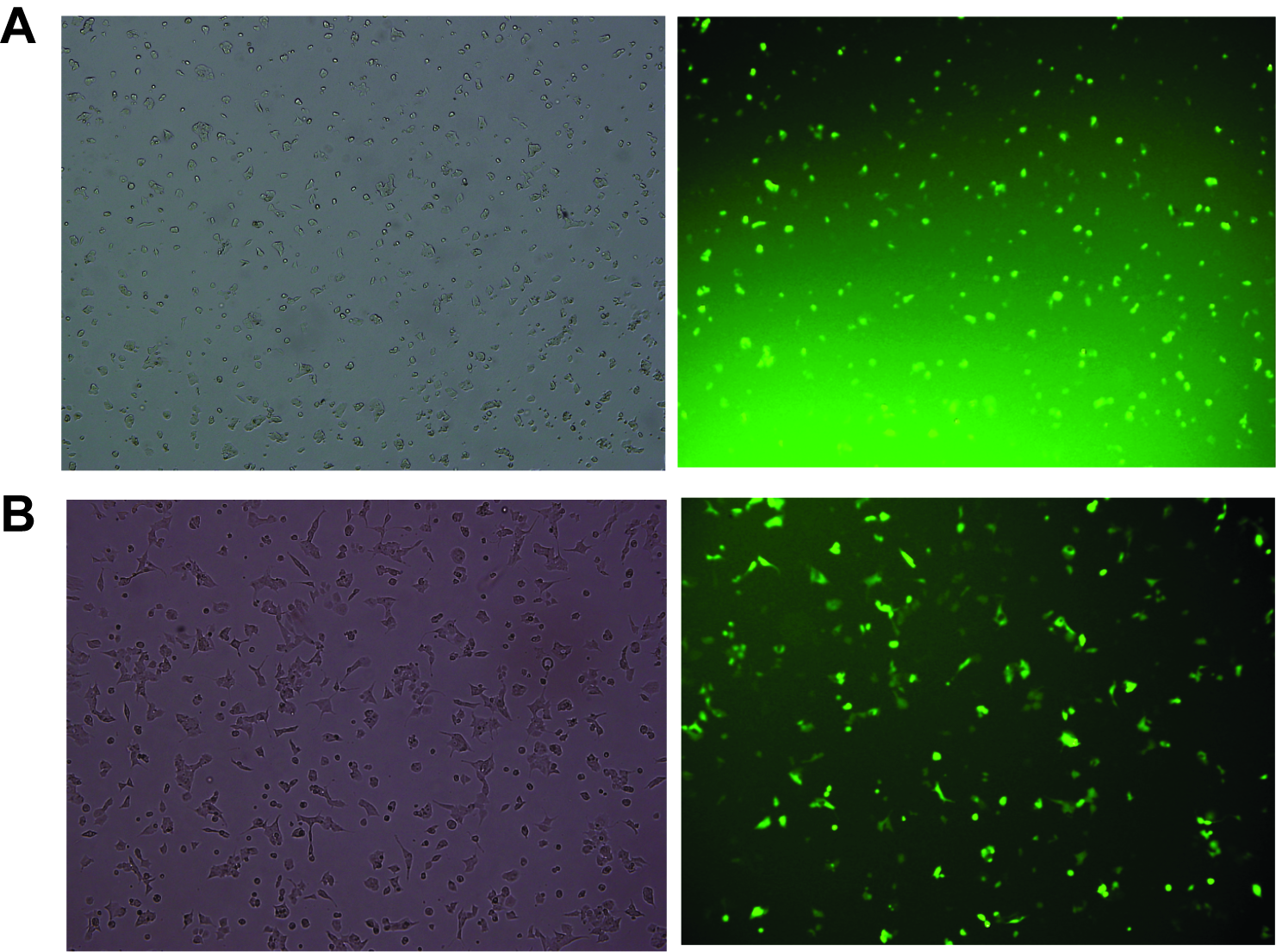
**

**Supplemental figure 1. Transfection efficiency of both panceatic β-cells. A:** transfection efficiency of HDAC4 (with GFP) to SJ-β cells (80-90%).**B**: transfection efficiency of HDAC4 to Min6 cells (>70%)


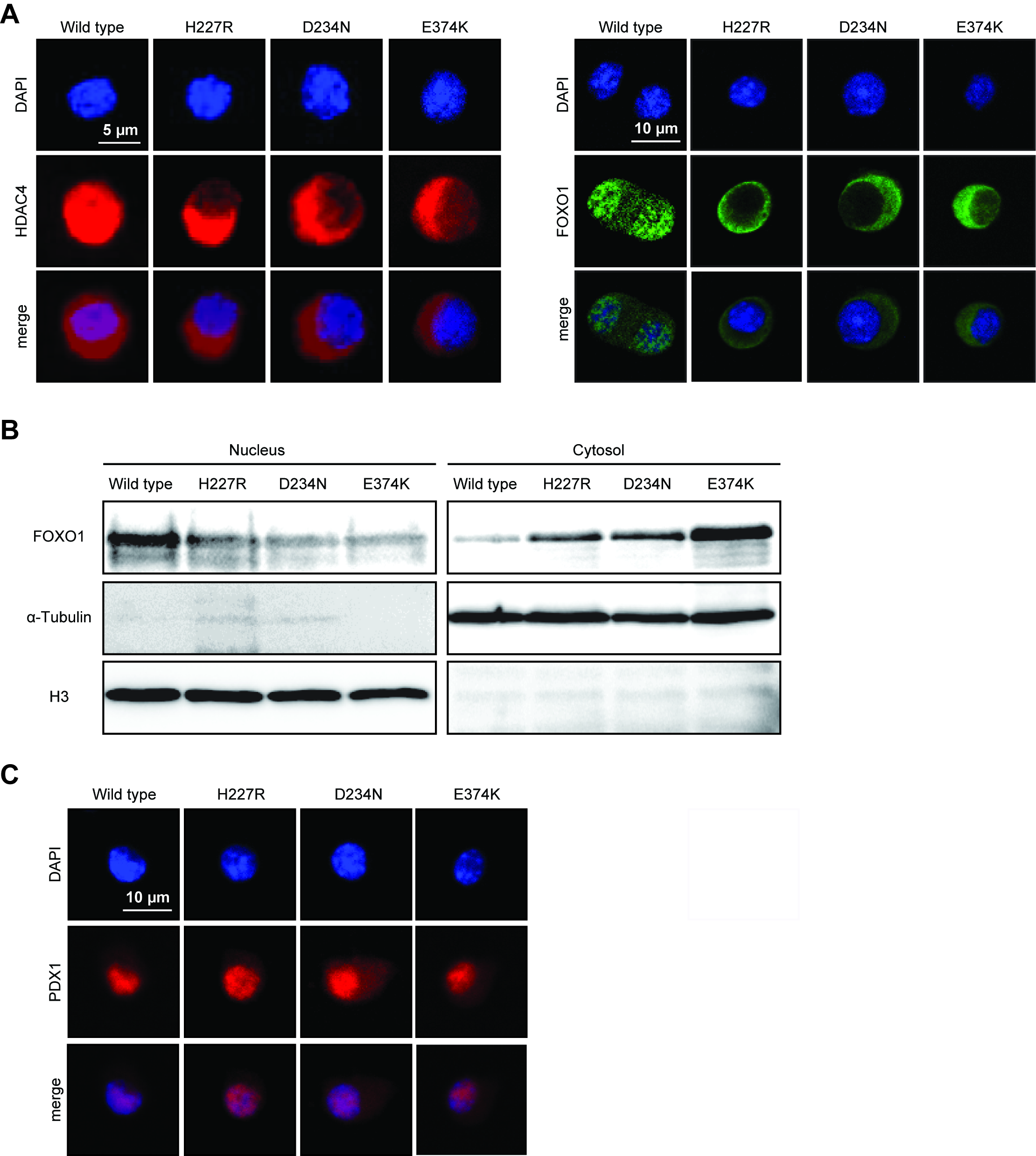


**Supplemental figure 2. *HDAC4* mutations export FoxO1, but not Pdx1 from nuleus to cytosol in SJ-β cells. A.** Immunostaining of DAPI (blue), HDAC4(red), and FoxO1 (green) in SJ-β cells that were transfected with wild-type (WT) or three mutated *HDAC4* (p.H227R, p.D234N, and p.E374K). Both HDAC4 (left) and FoxO1 (right) were translocated into the cell cytosol in the mutated *HDAC4* transfected cells compared to the wild-type of *HDAC4* transfected cells. **B.** Immunoblotting with antibodies against FoxO1, α-Tubulin, and Histone 3 (H3) of lysates separately extracted from nucleus (left) and cytosol (right) of SJ-β cells that were transfected with wild-type (WT) or three mutated *HDAC4* (H227R, D234N, and E374K).**C A.** Immunostaining of DAPI (blue), Pdx1(red) in SJ-β cells that were transfected with wild-type (WT) or three mutated *HDAC4* (p.H227R, p.D234N, and p.E374K). The cellular location of Pdx1 remains unchanging in the SJ-β cells despited the transfection of the *HDAC4* mutations.

**Supplemental Table 1. *De novo* variants detected from the exome sequencing which were predicted to be disease causing in the patient 1**

| **Chr** | **Pos (hg19)** | **REF/ALT** | **gene** | **PolyPhen2 score** | **SIFT**  **score** | **PhyloP score** | **MutationTaster score** | **GERP++ score** | **LRT score** |
| --- | --- | --- | --- | --- | --- | --- | --- | --- | --- |
| chr3 | 184.075.857 | T/C | *EIF2B5* | 0,97 | 0,01 | 0,996 | 1 | 4,1 | 1 |
| chr1 | 209.907.741 | C/T | *HSD11B1* | 1 | 0 | 0,999 | 0,995 | 5,28 | 1 |
| chr12 | 32.832.369 | G/T | *DNM1L* | 0,964 | 0,01 | 0,998 | 0,999 | 4,2 | 1 |
| chr2 | 240.078.401 | T/C | *HDAC4* | 0,879 | 0,43 | 0,995 | 0,988 | 4,02 | 0,999 |
| chr5 | 154.173.470 | A/T | *LARP1* | 0,941 | 0 | 0,998 | 0,973 | 5,37 | 1 |
| chr2 | 37.215.927 | G/A | *HEATR5B* | 0,996 | 0,15 | 0,999 | 0,996 | 5,18 | 1 |
| chr10 | 45.480.383 | C/T | *RASSF4* | 1 | 0 | 0,984 | 0,999 | 3,86 | 1 |
| chr13 | 39.264.467 | G/C | *FREM2* | 0,941 | 0,07 | 0,993 | 0,997 | 4,39 | 0,999 |

**Supplemental Table 2. *HDAC4* primers for gene screening and mutagenesis**

| **Primer name** | **Forward primer (5’to3’)** | **Reverse primer( 5’ to3’)** | **Product (bps)** |
| --- | --- | --- | --- |
| Exon2 | ACCGTCCCGGTACTTGTATG | CAAATCCAGAAAGCAAGCTG | 227 |
| Exon3 | CATGCATTTCATTGTTTGGG | GCCCTCTCTGCACTCCTC | 273 |
| Exon4 | AAGGTGGCCAGTGTGTTG | CGTCCCAACGCAGAAGG | 475 |
| Exon5 | CGGAATGGCCCTGACTTCTT | CTCACCTTCCGGAGTCCATG | 599 |
| Exon6 | CTTCCCTCCCTCTGTGGC | ATTCCTCACCCTCCTTCCC | 331 |
| Exon7 | GAAAGATCATCGTGTGTGGC | TAAGTGGAAGACAGCGGTGG | 306 |
| Exon8 | TCTCCCCTGCAGCAACAC | ACACGTGGGTTTTCAGAAGC | 364 |
| Exon9 | AAGCTAACGAGCAGGTCCG | AGTGAAGGGCAAGTGCAAAG | 314 |
| Exon10-11 | CAGTGCAGGTGTTTTCATCG | CAG ACA GAA ACC CAA ATC GC | 774 |
| Exon12 | AGCCAGCTCTGGGGCAC | CCTTAAGGTCAGAAAGGGGC | 478 |
| Exon13 | CAGAGGGTGGGGTTTGC | GACAGTGACCGCGCAAG | 466 |
| Exon14 | TATGGCCTGTGCGTGGAC | CCCTCCTCAGCCTCTGC | 397 |
| Exon15 | TCCTTCTAGGGAAGTGGTGG | GAACCACCTGCAAAACCAAC | 344 |
| Exon16 | CTTTTGGGCAATGGTTTCAG | TATAACCTGGCAGGCGACAC | 331 |
| Exon17 | AAGTGCCACTGAAGACCCC | TCACTTTGAGGGAAGCAAGG | 232 |
| Exon18 | CCTGGGGTTTCAGAAGCAG | CAGCCTGATGAGAGGGAGAC | 318 |
| Exon19 | CCTCCTGTGTAGCCCAACTC | CTCCCCGCAGTCACTCAC | 256 |
| Exon20 | GCTCTGCACTCACAGAGACC | AGCCCAGCTCCTCTGTCC | 298 |
| Exon21 | TAAGCACAACTGGGGTTGG | GAAGGCCGCACTCACTG | 321 |
| Exon22 | ACCAGGAGATCAGGACACG | CAGCCACAGACCAGTTTCAG | 347 |
| Exon23 | GCATTATCAATGCCAGAGGG | AAAAGGTGCCCTTCCTTATCTC | 322 |
| Exon24 | GTCTCGACACCCCAGCC | TTTCATGCTCTGGGGCTC | 335 |
| Exon25 | TGCTCAGGAAACAGTTGCAG | AGTGGGATGGAGAAGAGCTG | 282 |
| Exon26-27 | TCGTGTCTCTTCAAAGACGG | GCTCCAAGAGAGCCCCAC | 758 |
| H227R | tcgacctcctataaccgcccggtcctggg | cccaggaccgggcggttataggaggtcga |  |
| D234N | aagtcatctttggcgttgtacattcccaggaccg | cggtcctgggaatgtacaacgccaaagatgacttc |  |
| E374K | gggaagggtgagtctcttggcgtcctg | caggacgccaagagactcacccttccc |  |

**Supplemental Table 3. Mouse quantitative RT-PCR primers for the pancreatic ß cells**

| **Gene** | **Exons** | **Forward primer** | **Reverse primer** |
| --- | --- | --- | --- |
| *Ppib* | E3-E4 | GGAGATGGCACAGGAGGAAA | GTAGTGCTTCAGCTTGAAGTTC |
| *Pdx1* | E1-E2 | TGGATGAAATCCACCAAAGCT | GGGTTCCGCTGTGTAAGCA |
| *Neurod1* | E1 | CTCCAGGGTTATGAGATCGTC | ACAGACAACAGGTCAAGGCAT |
| *Foxa2* | E2-E3 | TGGGAGCCGTGAAGATGGAA | TGTGTTCATGCCATTCATCCCC |
| *Hnf1a* | E1-E2 | GCCGTGGTGGAGTCACTTCT | TGCAAGTACGACTTGACCATCTTC |
| *Hnf1b* | E4-E5 | TCCTCTCCACCCAACAAGATG | GACTGATTGTCGAAGAGGAAGTGA |
| *Hnf4a* | E3-E4 | GTTACTGCAGGCTTAAGAAGTGCTT | GGTCCCGCTCATTTTGGA |
| *Gck* | E4-E5 | CTTCACCTTCTCCTTCCCTGTAAG | GGCCTTGAAGCCCTTGGT |
| *Ins1* | E2 | GAAGTGGAGGACCCACAAGTG | CAACGCCAAGGTCTGAAGGT |
| *Ins2* | E2 | GCTGGCCCTGCTCTTCCT | AACCACAAAGGTGCTGCTTGA |
| *Slc2a2* | E9-E10 | TGTTTTTCTGCACCATCTTCATG | CGTAACTCATCCAGGCGAATTT |
| *MafA* | E1 | GCCACCACGTGCGCTTG | CCGCTTCTGTTTCAGTCGGAT |
| *Nkx6.1* | E1-E2 | CCATCTTCTGGCCCGGAGT | CTCCAGGGCGAAGATTTGCT |
| *Abcc8* | E11-E12 | TGCCGTCCTCATTACCTTTG | GGATGTGGAAGAGAGAGAGAGA |
| *Kcnj11* | E1 | GGACCTGGAGATCATTGTCATC | TAGAATCTCGTCAGCTAGGTAGG |
| *FoxO1* | E1-E2 | GTACGCCGACCTCATCACC | TCTGCACTCGAATAAACTTGC |
| *Hdac4* | E18-E20 | CCACGGGAGAGCTAAAGAATG | AACTGCCACGGAGTTAAAGTAG |

E: exon

**Supplemental Table 4. Antibodies for the immunochemistry, Western blot, and immunoprecipitation blot**

| **Antibody** | **Company** | **Catalogue Nr** |
| --- | --- | --- |
| Anti-FoxO1 | Cell Signalling Technology | #2880 |
| Anti-Pdx1 | Santa Cruz | sc-14664 |
| Anti HDAC4 | Cell signaling technology | #7628 |
| Anti- α-Tubulin | Sigma-Aldrich | #T6199 |
| Anti-total histone H3 (signaling, #4499) | Cell signaling technology | #4499 |
| Anti-acetylated lysine signaling#9441) | Cell signaling technology | #9441 |
| Anti- α-Tubulin for IP | Sigma-Aldrich | #T5168 |

**Web Resources**

The URLs for data presented herein are as follows:

gnomAD browser beta | [genome Aggregation Database](http://gnomad.broadinstitute.org/about): <http://gnomad.broadinstitute.org/>

ExAC Browser (Beta) | [Exome Aggregation Consortium](http://exac.broadinstitute.org/about): http://exac.broadinstitute.org/

UCSC Genome Browser: <http://genome.ucsc.edu>

National Center for Biotechnology Information (NCBI): <http://www.ncbi.nlm.nih.gov>

Picard-Tools: http://broadinstitute.github.io/picard/

Short Genetic Variations Database dbSNP137: <http://www.ncbi.nlm.nih.gov/SNP>,

**NHLBI GO Exome Sequencing Project:** <https://esp.gs.washington.edu/drupal/>

ENSEMBL: http://aug2010.archive.ensembl.org/index.html

1000 genomes project: [www.1000genomes.org](http://www.1000genomes.org)

PhastCons from alignment of 46 vertebrate species: <http://hgdownload-test.cse.ucsc.edu/goldenPath/hg19/phastCons46way>

SIFT: http://sift.jcvi.org

Polyphen-2: <http://genetics.bwh.harvard.edu/pph2>

Mutation Taster: [www.mutationtaster.org](http://www.mutationtaster.org)

GERP++: <http://mendel.stanford.edu/SidowLab/downloads/GERP/index.html>

LRT: http://www.genetics.wustl.edu/jflab/lrt_query.html

Endearvour: http://homes.esat.kuleuven.be/~bioiuser/endeavour/index.php

Sequence alignment: <http://www.uniprot.org/align/>

QuikChange Primer Design: http://www.genomics.agilent.com/primerDesignProgram.jsp
